# Evaluation of weight change in three different light cured composite restorative materials stored in water: An *in vitro* study

**DOI:** 10.4103/0972-0707.45248

**Published:** 2008

**Authors:** Mithra N Hegde, Basawaraj Biradar

**Affiliations:** Department of Conservative Dentistry and Endodontics, A. B. Shetty Memorial Institute of Dental Sciences, Mangalore, Karnataka, India

**Keywords:** Electrical analytical balance, micro-fine hybrid, nano-filled composite, water absorption, water storage, weight change

## Abstract

**Aims and Objectives:**

The objective of this in vitro study was to investigate whether weight gain in three different composites occurs due to water absorption, when stored in water.

**Materials and Methods:**

The composite restorative materials selected for this study included a micro-fine hybrid (Synergy) and two nano-filled composites (CERAM X duo and FILTEK Z 350). Twenty specimens of each material were fabricated with each composite material.– Group A: Filtek Z 350, Group B: Synergy, and Group C: CERAM X Duo. Then all the specimens were stored in 10 ml distilled water in test tubes, and then placed in an incubator at 37oC for six weeks. The weight changes of these specimens were measured daily for the first week, and, later, once a week, for the next five weeks, by using an electrical analytical balance.

**Results:**

The data were analyzed using one-way analysis of variance and Student ‘t’ test. There was a tendency for the weight of the specimens to increase with the passage of time, when stored in water. All the groups showed maximum amount of water absorption in the first week; then there was a gradual decrease in the water absorption, from the second to the sixth week. Synergy showed the maximum amount of water absorption in the first week, as compared to FILTEK Z 350 and Ceram X Duo. However, FILTEK Z 350 showed the maximum amount of water absorption from the second week to the sixth week, as compared to Ceram X Duo and Synergy.

**Conclusion:**

All composite restorative materials absorb some amount of water. The water absorption of the composite may decrease the physical and mechanical properties of the composites; hence, it is necessary to consider the type of the material before starting treatment.

## INTRODUCTION

In recent years, the attractiveness of tooth colored restorations has stimulated research in this particular area of operative dentistry. Patients are increasingly demanding esthetic restorations not only in the anterior teeth, but also in the posterior teeth. Dental material composites being used widely today are chosen not only for their esthetic properties, but also for the ability to adhere to tooth substance.[1,2]

Mechanical properties of composites are influenced not only by their chemical composition but also by the environment to which they are exposed. The corrosion process promoted by water and the presence of constant load on the surface of resin are responsible for the appearance and propagation of interfacial debonding, matrix cracking, superficial flaws, filler dissolution and filler particle dislodgement.[3]

Nanotechnology, also known as molecular nanotechnology or molecular engineering, is the production of functional material and structures in the range of 0.1 to 100 nanometers by various physical and chemical methods. The intense interest in nanomaterial is to provide a dramatic improvement in electrical, chemical, mechanical and optical properties.[4]

The problem associated with these restorative materials is water absorption, as they are continuously bathed in saliva. For resin based composite materials, water absorption may induce weakening of the matrix and breakdown of resin filler interface. It is also expected that absorption of water will be accompanied by hygroscopic expansion of the composite, which may be able to compensate for the effect of polymerization shrinkage and relieve stresses.[1]

The dimensional changes in composite restorative materials placed in the cavity are the result of shrinkage of resin monomer during polymerization. Shrinkage is compensated for by the expansion resulting from the water absorption of the set resin. This fact has drawn much attention, with regard to the adaptation of the composite to the dental cavity walls.[2,5,6]

Water sorption actually increases with cross linker concentration, suggesting that the chemical nature of cross linking agent may supercede the effect of higher molecular density; a high level of porosity or microvoids has also been shown to facilitate fluid transport into and out of the polymer.

The aim of this study is to evaluate the weight change of three different light cured composite restorative materials stored in water.

## MATERIALS AND METHODS

Twenty specimens from each composite material were prepared, using brass mold (6 mm diameter × 2 mm height). The composite material was covered with acetate strips and compressed between two glass slabs, to remove voids and extrude excess composite material. The composite was then light cured through the acetate strip for 40 seconds by using quartz tungsten halogen light curing unit (QHL-75, Dentsply). The light curing unit was held at a distance of 1 cm from the specimen and curing was done at an intensity of 450 mW/cm^2^. The tip diameter of the light curing unit was 11 mm.

Following light curing, the specimens were removed from the mold and finished with carborundum paper and later polished with coarse, medium and fine Sof-lex discs (3M ESPE), in respectively. The specimens were then weighed using an electrical analytical balance (DANVER INSTRUMENT) and placed in a test tube containing 10 ml distilled water, measured using a measuring jar (Borosil). The specimens were sealed in the test tube with cotton pellets and placed in an incubator at 37°C, for six weeks.

After 24 hours, the specimens were removed and placed on filter paper (Whatman) for a period of 1 min, to drain the excess water and then weighed accurately, using an electrical analytical balance.

After weighing the specimens, they were transferred to the test tubes filled with 10 ml of fresh distilled water, measured using a measuring jar.

The procedure was repeated every day for the first week; and, later, once a week for the next five weeks.

### Analysis of data

The data obtained were analyzed statistically, using Analysis of variance (ANOVA) and Student t test.

## RESULTS

The data were analyzed using the multivariate approach of repeated measurements. The techniques used were one-way analysis of variance (ANOVA) and Student ‘t’ test, with the help of SPSS Version 13.00.

All the groups showed maximum amount of water absorption in the first week; then there was a gradual decrease in the water absorption from the second to the sixth week [Table 1-5].

**Table 1:** Materials used

Materials used	Manufacturer	Composite type	Matrix
Filtek Z 350 (Group A)	3M ESPE	Nano-composite	Nano-composite, universal restorative material. Aggregated zirconia/silica cluster filler, with an average particle size of 0.6 – 1.4 micron
Synergy (Group B)	Coltene whaledent	Nano hybrid	Micro-fine hybrid BisGMA, BisEMA, TEGDMA, Strontium glass, barium glass, amorphous silica
Ceram X duo (Group C)	Dentsply	Nano-composite	nano-ceramic methacrylate modified polysiloxane, dimethacrylate resin, fluorescence pigment, camphoroquinone.

**Table 2:** The mean weights of three composite specimens, measured daily during the first week of the observation

	No. of observations	Group A	Group B	Group C
Initial	20	929.2 (28.140)	910.7 (18.979)	905.6 (15.806)
First day	20	932.5 (26.963)	916.7 (19.074)	908.3 (15.267)
Second day	20	933.8 (27.976)	919.1 (16.368)	910.0 (15.499)
Third day	20	936.2 (26.246)	921.2 (15.161)	911.7 (115.291)
Fourth day	20	938.0 (23.576)	922.8 (15.087)	913.5 (14.855)
Fifth day	20	940.4 (25.124)	924.4 (15.916)	915.5 (15.157)
Sixth day	20	943.8 (25.614)	928.0 (14.706)	917.5 (14.652)

(Standard deviations are given within brackets)

**Table 3:** The mean weights of the three composite specimens, measured daily during the entire period of the observation

	No. of observations	Group A	Group B	Group C
Initial	20	929.2 (28.140)	910.7 (18.979)	905.6 (15.806)
First week	20	943.8 (25.614)	928.0 (14.706)	917.5 (14.652)
Second week	20	945.4 (25.488)	929.5 (14.580)	919.4 (13.808)
Third week	20	947.7 (26.725)	930.6 (14.303)	920.3 (113.632
Fourth week	20	950.2 (26.998)	931.2 (14.722)	922.1 (12.377)
Fifth week	20	953.3 (28.507)	932.8 (14.388)	923.4 (12.445)
Sixth week	20	955.3 (30.479)	934.4 (13.936)	924.5 (12.441)

(Standard deviations are given within brackets)

**Table 4:** The mean weight changes in the three groups during the first week of observation (Initial day to sixth day)

Groups	Paired difference					*P*
		
	Mean difference	Std. error	95% confidence interval for the mean difference		t	
					
			Lower limit	Upper limit		
Group A	14.600	2.614	9.129	20.071	5.585	<0.001
Group B	17.350	2.141	12.868	21.832	8.102	<0.001
Group C	11.900	1.499	14.184	23.576	7.939	<0.001

**Table 5:** The mean weight changes in the three groups during the entire period of the observation

Groups	Paired difference					*P*
		
	Mean difference	Std. error	95% confidence interval for the mean difference		t	
					
			Lower limit	Upper limit		
Group A	26.150	3.565	18.688	33.612	5.585	<0.001
Group B	23.700	2.090	19.325	28.075	8.102	<0.001
Group C	18.850	2.229	14.184	23.516	7.939	<0.001

(Initial day to sixth week)

Among the groups, Synergy showed the maximum amount of water absorption in the first week, as compared to FILTEK Z 350 and CERAM X Duo. However, FILTEK Z 350 showed the maximum amount of water absorption from the second week to the sixth week, as compared to CERAM X Duo and Synergy.

There was no significant difference noted among the materials (*p*>0.005). As a result, the difference between the groups was not compared.

## DISCUSSION

Weight change in water was evaluated because saliva is a dilute fluid comprising 99% water. The concentrations of dissolved solids (organic or inorganic) are characterized by wide variations, both between individual and within a single individual. Due to this variation, water was used as the test standard.[1]

Brass was chosen for this study because many of its physical properties are similar to those of the tooth substance. For example, the Young's modulus of brass is very close to that of enamel, while its hardness lies in between the hardness of enamel and dentin. Its co-efficient of thermal expansion is similar to that attributed to tooth structure.[2]

Quartz tungsten halogen light curing unit, with an intensity of 450 mW/cm^2^ and wavelength between 400 and 500 nm, which was sufficient to cure composite specimen's up to a depth of 2 mm, was used.

Acetate strips were used to prevent the formation of oxygen-inhibited layer on the surface of the composite.[7]

The factors which affect the amount of water absorption of the composite restoration materials are resin content, filler content and the coupling agent. The more the filler content, the lesser will be the water absorption, the proper the bonding of the coupling agent , and the lesser the water absorption.[1]

In the present study, the weight change of the specimen was measured according to the ISO 4049 (International Organization of Standardization) original plan[8] (1985) and water solubility of the specimen was determined as per ADA specification no. 8 (1978).[9]

This study showed the maximum amount of water absorption in the first week of the experiment. The dimensional changes in composite restorative materials in the first week were the result of the shrinkage of the resin monomer during polymerization in the first week. Shrinkage was compensated for by the expansion resulting from the water absorption of set resin. This fact has drawn much attention, with regard to the adaptation of the composite to the dental cavity walls.[2,4,5]

A study done by Iwami and Yamamoto also showed the maximum amount of water absorption in the first week of the experiment.[10] A study done by Filiz and Yalgin also showed the maximum amount of water absorption in the first week of the experiment.[1]

This study showed that Synergy absorbs the maximum amount of water, as compared to Filtek Z 350 and Ceram X Duo. This is because Synergy contains increased resin to filler ratio.[1] However, in this study, the focus was only on the relationship between immersion time, water absorption of the resin and thickness of the specimen. In other words, weight loss due to dissolution was not included in the measurement. The diffusion co-efficient and the thickness of the specimen were affected by the amount of water absorption.[2]

In this study, all materials showed a 90% increase of final volumetric expansion and change in weight within seven days. Thereafter, there was a slower and more gradual increase in the volume and weight. This two stage expansion may be caused due to the hydrolytic degradation of monomer bonds or stretching of these bonds beyond their elastic limit, causing them to rupture.

The study done by Iwami and Yamamoto also showed that more than 90% of water absorption occurred in first week.[9]

The increase in the dimension shown by the materials may be beneficial in relieving some of internal polymerization shrinkage stresses and it may increase the longevity of the adhesive union to surrounding tooth.[11]

Studies on the amount of weight loss due to dissolution, diffusion co-efficient, thickness of the specimen, and changes in physical and mechanical properties are further required before drawing any conclusive clinical assessment.

## CONCLUSION

The present *in vitro* study evaluated the weight change of microfine hybrid (Synergy), and two different nano-filled (Filtek Z 350 and Ceram X Duo) composite restorative materials.

The following conclusions were drawn:

1. All the groups showed some amount of weight gain due to water absorption.
2. All the groups showed the maximum amount of weight gain in the first week and slowly decreased from the second to the sixth week
3. Synergy showed the maximum amount of water absorption in the first week, as compared to Filtek Z 350 and Ceram X Duo.
4. Filtek Z 350 showed the maximum amount of water absorption from the second to the sixth week, as compared to Ceram X Duo and Synergy.
